# Reductive stress on life span extension in *C. elegans*

**DOI:** 10.1186/1756-0500-1-19

**Published:** 2008-06-04

**Authors:** Markus Ralser, Ivor J Benjamin

**Affiliations:** 1 Max Planck Institute for Molecular Genetics, Ihnestrasse 73, 14195 Berlin, Germany; 2 University of Utah School of Medicine, 30 North 1900 East, Salt Lake City, UT 84132, USA

## Abstract

Recently, Schulz and colleagues have contributed to the ongoing controversy on the unproven role of oxidative stress in the aging process in their well-performed study 'Glucose restriction extends *Caenorhabditis elegans *life span by inducing mitochondrial respiration and increasing oxidative stress' (Cell Metab 2007, 6: 280–293). Here, we suggest an alternative hypothesis that reductive stress can prevent calorie-restriction induced life span extension. We draw attention to this condition as an explanation for some contradictory observations including the deleterious effects from antioxidants.

## Discussion

How – or if at all – free radicals, oxidants, and oxidative stress contribute to the aging process is a fundamental, voluminous but still controversial research topic. In a recent Edition of Cell Metabolism, Schulz and colleagues report their important and well-controlled observations of 2-Deoxy-D-Glucose (DOG or 2-DG)-induced caloric restriction on life span in *C. elegans *[1]. The authors observe that treatment with 2-DG (a glucose analogue and inhibitor of two glycolytic enzymes) increases *C. elegans' *life span as well as mitochondrial respiration, resulting in increased generation of reactive oxygen species (ROS). However, when animals were treated with antioxidants such as N-acetylcysteine (NAC, a precursor of reduced glutathione (GSH)), the effects of 2-DG on life span extension were abolished. To explain these intriguing findings, these authors suggested that glucose restriction induces mild oxidative stress originating from mitochondria and increases protective mechanisms, termed 'mitohormesis,' resulting in the increased longevity of the model organism and extension of *C. elegans *life span (see [2] for a review). Notwithstanding, the relationship between hormesis and the 'oxidative-stress' theory of ageing remains uncertain (see [3] for a review).

Although the oxidative stress theory of aging is widely accepted, the mechanisms underlying mammalian aging remains poorly understood as several animals models in which oxidative stress is enhanced have neither shortened life span nor recapitulated aging phenotypes in mice [4].

First, cellular redox homeostasis in organisms is highly dependent on both enzymatic and non-enzymatic pathways. Cells exposed to oxidative stress conditions have increased activity of the pentose phosphate pathway, which replenishes cellular reduction of NADP^+ ^to NADPH [5-7]. For example, in response to ROS production, reducing equivalents in the form of NADPH and GSH, both which serve to neutralize pro-oxidants (i.e., anti-reductant), are generated by the anti-oxidative machinery to restore redox homeostasis. This mechanism allows the cell to restore the redox state under the aforementioned oxidative stress conditions, principally from high levels of (reduced) NADPH and/or recycling the synthesis of glutathione (GSH), which are required by the cellular antioxidant systems [8].

However, *reductive stress *in the form increased GSH production has been genetically and causally linked to dysregulation of antioxidative pathways in mice [9,10]. Indeed, recent results show deleterious effects of reductive stress in different organisms, including reduced life span of yeast and *C. elegans *and age-related protein aggregation disease in mice [7,10], unpublished studies from IJB). How does reductive stress prevent life span extension in mammals? By presenting partially understood mechanisms, we hypothesize that activation of compensatory pathways such as activated by the glucose 6-phosphate dehydrogenase (G6PD), the rate-limiting enzyme of the anaerobic pentose phosphate shunt, escapes feedback control, triggering excess amounts of reducing equivalents into deleterious zone. In conditions promoting ROS production without consumption of the newly produced reducing equivalents (a situation that could be provoked by combining pro- and anti-oxidative treatments), we envision plieotrophic effects on gene expression, protein folding and metabolic processes.

If ROS production is not a significant cause of aging in *C. elegans*, further studies are needed to clarify the alternative roles of glucose restriction and 2-DG treatment on generation of reducing equivalents. For example, we suggest that the impact of antioxidant treatment on the ageing process needs to be clarified at the level of glutathione synthesis (i.e. gamma glutathione synthetase) and recycling (e.g., glutathione reductase). Cellular and molecular studies such as gene deletion or knock-down of antioxidant and pro-oxidant systems, careful titration of oxidants and reductants might be combined with biochemical analyses of the cellular redox state, especially of the NAD(H), NADP(H) and GS(S)H pools.

Notwithstanding, we fully agree with and wish to highlight the authors' conclusion that the rationale for the use of antioxidants in the arsenal against aging and health maintenance and/or prevention needs to be revisited.

## Competing interests

The authors declare that they have no competing interests.

## Authors' contributions

MR and IJB discussed the topic and wrote the manuscript.
